# Physical Exercise and Myokines: Relationships with Sarcopenia and Cardiovascular Complications

**DOI:** 10.3390/ijms21103607

**Published:** 2020-05-20

**Authors:** Sandra Maria Barbalho, Uri Adrian Prync Flato, Ricardo José Tofano, Ricardo de Alvares Goulart, Elen Landgraf Guiguer, Cláudia Rucco P. Detregiachi, Daniela Vieira Buchaim, Adriano Cressoni Araújo, Rogério Leone Buchaim, Fábio Tadeu Rodrigues Reina, Piero Biteli, Daniela O. B. Rodrigues Reina, Marcelo Dib Bechara

**Affiliations:** 1 Postgraduate Program in Structural and Functional Interactions in Rehabilitation, University of Marilia (UNIMAR), Avenue Hygino Muzzy Filho, 1001, Marília 17525-902, São Paulo, Brazil; uriflato@gmail.com (U.A.P.F.); rtofano@uol.com.br (R.J.T.); ricardogoulartmed@hotmail.com (R.d.A.G.); elguiguer@gmail.com (E.L.G.); claurucco@gmail.com (C.R.P.D.); danielaortega@ig.com.br (D.V.B.); adrianocressoniaraujo@yahoo.com.br (A.C.A.); rogerioleonibuchaim@unimar.br (R.L.B.); fabioreina@unimar.br (F.T.R.R.); pbiteli@icloud.com (P.B.); danielareina@unimar.br (D.O.B.R.R.); 2 School of Medicine, University of Marília (UNIMAR), Avenida Higino Muzzi Filho, 1001, Marília 17506-000, São Paulo, Brazil; dib.marcelo1@gmail.com; 3 Department of Biochemistry and Nutrition, Food Technology School, Marília 17525-902, São Paulo, Brazil; 4 Medical School, University Center of Adamantina (UniFAI), Adamantina 17800-000, São Paulo, Brazil; 5 Department of Biological Sciences, Bauru School of Dentistry, University of São Paulo (FOB–USP), Alameda Doutor Octávio Pinheiro Brisolla, 9-75, Bauru 17012901, São Paulo, Brazil

**Keywords:** sarcopenia, myokines, cardiovascular diseases

## Abstract

Skeletal muscle is capable of secreting different factors in order to communicate with other tissues. These mediators, the myokines, show potentially far-reaching effects on non-muscle tissues and can provide a molecular interaction between muscle and body physiology. Sarcopenia is a chronic degenerative neuromuscular disease closely related to cardiomyopathy and chronic heart failure, which influences the production and release of myokines. Our objective was to explore the relationship between myokines, sarcopenia, and cardiovascular diseases (CVD). The autocrine, paracrine, and endocrine actions of myokines include regulation of energy expenditure, insulin sensitivity, lipolysis, free fatty acid oxidation, adipocyte browning, glycogenolysis, glycogenesis, and general metabolism. A sedentary lifestyle accelerates the aging process and is a risk factor for developing sarcopenia, metabolic syndrome, and CVD. Increased adipose tissue resulting from the decrease in muscle mass in patients with sarcopenia may also be involved in the pathology of CVD. Myokines are protagonists in the complex condition of sarcopenia, which is associated with adverse clinical outcomes in patients with CVD. The discovery of new pathways and the link between myokines and CVD remain a cornerstone toward multifaceted interventions and perhaps the minimization of the damage resulting from muscle loss induced by factors such as atherosclerosis.

## 1. Introduction

Skeletal muscle is the most massive protein reserve in the human body, the primary site of glucose metabolism regulation, and the main energy consumer. In addition to glycogenolysis, skeletal muscle is degraded during starvation and produces amino acids and lactate that are utilized in hepatic and renal gluconeogenesis. Muscles can also release microRNA. Furthermore, skeletal muscle is capable of secreting different factors in order to communicate with other tissues and of releasing autocrine, paracrine, and endocrine mediators termed myokines. These mediators show potentially far-reaching effects on non-muscle tissues and can provide a molecular interaction between muscle function and body physiology [1,2,3].

Both exercise and muscle disuse can lead to numerous physiological adaptations by augmenting or reducing the release of myokines. Among the many changes that accompany the process of human aging is muscle loss, consisting in a progressive decrease of skeletal muscle mass that may reach approximately 6% per decade after mid-life [4,5,6].

Several studies show that there is a significant difference in muscle mass between individuals with peak muscle mass and those undergoing muscle loss over time. The term that refers to this process is *sarcopenia, which derives from sarx* (which means “flesh”) and -*penia* (which implies “loss”) [7,8].

Metabolic and cardiovascular risks are also closely related to aging, and cardiovascular diseases (CVD) are an important worldwide cause of disability and mortality. Although the pathways have not yet been completely elucidated, numerous authors have shown an association between sarcopenia and cardiovascular complications [9,10,11,12,13]. For these reason, the purpose of this review is to evaluate the relationship between myokines, sarcopenia, and CVD.

## 2. Discussion

### 2.1. Myokines

Under physical exercise conditions, muscles can produce and release molecules, cytokines, or signaling peptides, named myokines. These molecules can exert paracrine and endocrine actions. Not all of them are exclusively produced by skeletal muscle, as they can also be released by other cells, such as adipose tissue (adipomyokines). Nevertheless, skeletal muscle is possibly the primary source of most myokines, since it represents over 30% of human body mass [14,15,16].

Physical exercise has positive effects on the balance between anti- and pro-inflammatory mediators. The release of the latter is related to a sedentary lifestyle. Inflammation enhances sarcopenia and accumulation of fat in skeletal muscle in a vicious circle, reducing muscle strength and favoring physical inactivity [17,18]. Besides that, visceral fat accumulation is a risk factor for chronic degenerative diseases, such as obesity, type 2 diabetes mellitus, CVD, cancer, dementia, and depression. This complex scenario characterized by physical inactivity and associated diseases is named *the diseasome of physical inactivity* [19].

Aerobic exercise can activate peroxisome proliferator-activated receptor γ coactivator 1-α (PGC1-α) that induces mitochondrial biogenesis and inhibits forkhead box O3 (FoxO3), which is important in the regulation of protein metabolism. Moreover, anaerobic exercise stimulates the production of myofibrillar proteins through the stimulation of the phosphoinositide 3-kinase/protein kinase B (PI3K/AKT) pathway and increased production of insulin-like growth factor-1 (IGF-1). These processes avoid muscle wasting by strengthening protein synthesis and inhibiting FoxO3 through AKT-mediated phosphorylation [20,21].

Myokines have been associated with the advantageous effects of regular exercise on health. Indeed, these muscular mediators are plausibly necessary for whole-body homeostasis, including metabolic reactions in cardiovascular, kidney, bone, and hepatic tissues [14,22,23,24,25].

There are about 3000 myokines that are regulated in response to muscle contraction and differentiation [15]. IL-6 was the first described myokine and can show anti-inflammatory properties in mammals. Some of the other myokines are apelin, brain-derived neurotrophic factor (BDNF), angiopoietin-like 4 (ANGPTL4), BAIBA (β-aminoisobutyric acid, a non-protein amino acid), fibroblast growth factor 21 (FGF-21), chemokine (C–C motif) ligand-2 (CCL-2) (also called monocyte chemoattractant protein-1 (MCP-1)), chemokine (C–X3–C motif) ligand 1 (CX3CL1) (also called fractalkine (FKN)), irisin, leukemia inhibitory factor (LIF), interleukin-6 (IL-6), IL-7, IL-8, IL-15, myostatin, meteorin-like protein (Metrnl), and secreted protein acidic and rich in cysteine (SPARC) [26,27,28].

Different types of muscle fibers release different myokines. Glycolytic fibers mainly produce myokines such as angiogenin, musclin, and osteoprotegerin, while oxidative fibres mainly produce myonectin and irisin. Interestingly, some myokines are induced by exercise through specific types of physical activity [29,30,31].

Myokines regulate several processes associated with physical frailty, including muscle dynapenia (age-related reduction in muscle strength), wasting, and slowness. The autocrine, paracrine, and endocrine actions of myokines include the regulation of energy expenditure, insulin sensitivity, muscle physiology, lipid metabolism (lipolysis, free fatty acid oxidation, and adipocyte browning), liver function (glycogenolysis and glycogenesis regulation), and metabolism [32,33,34,35]. Below, we will briefly discuss the role of some relevant/better-known myokines.

Myostatin (also termed growth and differentiation factor-8 (GDF-8)) is a negative regulator of muscle mass, impairing muscle synthesis and augmenting muscle catabolism. The activation of myostatin upregulates activin type II and type I receptors, which leads to the phosphorylation and activation of small mothers against decapentaplegic (SMAD) proteins, mostly SMAD-2 and SMAD-3, forming a complex with SMAD-4 that results in the transcription of target catabolic genes. Beyond that, it can promote muscle loss due to the impairment of the activation of satellite cells, Akt, and myogenic factors, as well to the stimulation of the ubiquitin–proteasome system (UPS). This myokine is a critical down-regulator of skeletal muscle and it possibly exhibits anti-anabolic effects on bone [36,37].

Since myostatin is related to the down-regulation of skeletal muscle growth, its inhibition may be a strategy for the treatment of muscle disorders, such as disuse muscle atrophy, sarcopenia, muscular dystrophy, cachexia, and cancer. Genetic defects causing decreased myostatin production result in muscle hypertrophy, while the overexpression of this myokine induces cachexia [38].

Follistatin is a myostatin-binding protein that is capable of inhibiting myostatin activity leading to muscle growth [39]. The plasma levels of follistatin increase with exercise. This myokine is also secreted by the liver; hence, it is also classified as a hepatokine. Follistatin regulates muscle growth, modulates pancreatic cell function and survival, and regulates leukocyte cell-derived chemotaxin 2 (LECT2), which is capable of promoting insulin resistance in skeletal muscle and adipose tissue [40].

Decorin expression is especially augmented after acute and chronic exercise and in contracting myotubes. It may also work as a counter-regulator of myostatin, inhibiting the activation of the SMAD-2/3 complex, possibly decreasing the degradation of muscle proteins [41,42,43]. It exhibits powerful anti-inflammation, antioxidant, antifibrotic, and antiangiogenic properties and regulates the differentiation, proliferation, and apoptosis of cells. Currently, decorin is considered a potential therapeutic target for both cachexia and cancer [44,45].

Another exercise-induced myokine is musclin, which is also expressed in bone, brown adipose tissue, and spleen. It seems it is involved in suppressing processes causing heart failure after myocardial infarction and in the modulation of bone mass. Besides that, its administration might be beneficial to individuals with cancer who are not able to exercise and are at higher risk for cachexia [46].

Apelin is related to the induction of mitochondriogenesis and can reduce inflammation, stimulate regenerative properties, and avoid age-associated muscle wasting. Likewise, apelin plays an essential role in many physiological and pathophysiological processes, such as metabolism, cardiac contractility, angiogenesis, regulation of blood pressure, inflammation, cell proliferation, and apoptosis [47,48,49]. During the aging process, the synthesis of apelin by skeletal muscle is decreased, and its plasma levels also decrease. On the other hand, aged animals treated with a daily injection of apelin showed improvement of muscle capacity and myofiber hypertrophy [48].

Myonectin is associated with lipid metabolism and nutritional status and is capable of activating AKT, insulin receptor substrate-1 (IRS-1), and mammalian target of rapamycin (mTOR). Moreover, it suppresses the transcription of autophagy genes and can act alone or in synergy with the activation of the IGF-1/PI3K/Akt/mTOR pathway [50]. Possibly, myonectin shows cardioprotective effects and can partly mediate the cardiovascular advantage of endurance exercise, which suggests that this myokine may play a role in the prevention and treatment of CVD that are ameliorated by physical exercise [51].

Irisin is a proteolytic product of fibronectin type III domain-containing 5 and is secreted after exercise in both humans and mice. In mice, this myokine has been shown to cause the trans-differentiation of white adipose tissue into brown adipose tissue (browning); it can also play a beneficial role in preventing/improving insulin resistance, metabolic syndrome, and CVD in humans [52]. Besides that, irisin increases the expression of uncoupling protein-1 (UCP-1), which is associated with the “browning” of subcutaneous white adipose tissue, induces an increase in energy expenditure, the improvement of insulin resistance, and possibly favors weight loss. Irisin increases the cortical bone mass due to the modulation of osteogenesis-stimulating factors. It may also promote the proliferation and differentiation of osteoblasts and bone marrow, decrease osteoclast formation, and increase bone mineral density. Irisin is termed an adipo-myokine, because it can be produced by white adipose and muscle tissue in response to exercise [53,54].

Irisin is an independent parameter associated with sarcopenia and carotid atherosclerosis [24] in dialysis patients, which suggests its significant role in CVD, at least in part, independently of inflammation that occurs in these patients [55].

Pathological or age-related sarco–osteoporosis (the combination of sarcopenia and osteoporosis) is crucial for human health and is related to an augmented risk of fracture in patients. Currently, the therapeutic approaches for the prevention of fractures are mainly focused on bone, but the increasing understanding of the muscle–bone crosstalk will probably encourage a change of paradigm, as shown in the study of Drei et al. [56]. This implies that novel therapeutic approaches should target both bone and muscle. As irisin is involved in both sarcopenia and osteoporosis, it may be a therapeutic target [57]. IL-6 is known as a pro-inflammatory cytokine and can be expressed by several cells, such as macrophages, fibroblasts, and vascular endothelial cells. It can stimulate bone resorption and increases osteoblastic differentiation. After exercise, the plasma levels of IL-6 can increase up to 100-fold, although this increase does not seem to be linear over time. The increase in IL-6 levels after exercise is followed by the incremented expression of IL-10 and IL-1 receptor antagonist. This chronic response constitutes an anti-inflammatory environment in response to the increase in circulating IL-6 induced by exercise. Furthermore, under specific conditions, IL-6 can inhibit the production of TNF-α [58,59,60].

β-amino-isobutyric acids (BAIBAs) are metabolites of valine and thymine and are produced primarily by muscles during physical exercise. In vitro studies show that BAIBA is related to the upregulation of both brown and beige adipocytes markers (e.g., UCP-1 and CIDEA), suggesting it can induce browning of white adipocytes and thermogenesis. BAIBA is also related to decreasing lipogenesis in white adipose tissue, reducing inflammation, and to insulin resistance [61].

**Figure 1:**
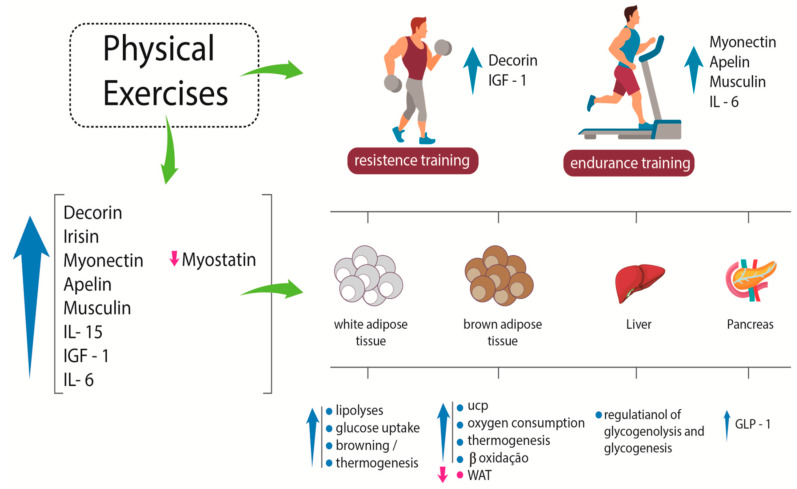
Systemic effects of some myokines. After resistance exercise, specific myokines, such as decorin and IGF-1, are released; after endurance training, other specific myokines, such as myonectin, apelin, musclin, and IL-6, are produced and play different roles in different tissues. IGF-1: insulin-like growth factor-1; IL-6: interleukin-6; IL-15: interleukin-15; UCP: uncoupling protein; WAT: white adipose tissue; GLP-1: glucagon-like peptide-1. Up arrows indicate increase; down arrows indicate reduction.

BDNF is another myokine whose concentration increases after physical exercise. It plays an essential role in regulating the growth, survival, and maintenance of neurons. Therefore, it is related to learning and memory. BDNF mRNA increases in human skeletal muscle after physical exercise, but unlike other molecules, this myokine is not released into the circulation. Its action is associated with an increase in fat oxidation due to the stimulation of AMPK, resulting in a decrease of adipose tissue bulk [36]. Figure 1 shows some effects of myokines on white adipose tissue, brown adipose tissue, liver, and pancreas.

### 2.2. Sarcopenia

Skeletal muscle is very plastic, since it can be modified/enlarged in response to exercise or can be substantially reduced in catabolic conditions, such as aging, or diseases, such as diabetes, cancer, cardiovascular diseases, chronic obstructive pulmonary disease, and infectious diseases. It is the major reservoir of proteins, but in catabolic condition, it can be reduced, leading to cachexia and death [15].

Sarcopenia is a chronic degenerative neuromuscular disease and a debilitating condition related to aging, which is characterized by the progressive loss of skeletal muscle, strength, and function. It is related to an increase in the risks of falls, fractures, and poor quality of life, which results in disability, frailty, and high mortality. Furthermore, sarcopenia is also related to several other health conditions, such as cognitive impairment, cardiac and respiratory disease, and osteoporosis. It is considered a public health problem, in light of its possible adverse outcomes [39,62,63].

The decrease in muscle size occurs because of a reduction in cell size (loss of proteins, organelles, and cytosol) driven by protein catabolism through amplified lysosomal and proteasomal functions activated by FoxO3-dependent transcription and reduced protein production upregulated by the IGF-1/PI3K/AKT pathway. This process also causes a reduction of mitochondria and, consequently, a reduction in energy production that leads to fatigue [64]. FOXO3a increases the expression of human ferritin and upregulates iron transport, leading to iron accumulation, which may negatively affect the production of myokines, and enhances motor neuron vulnerability to degeneration [65].

The interpretation of the measurements to evaluate sarcopenia is challenging, since they parameters measured are not muscle-specific or sensitive to the etiology. Also, there are confounding elements, such as fitness level and presence of pain and depression. Furthermore, individuals in the later stages of sarcopenia might eventually not be able to perform these tests. For these reasons, effective diagnosis, treatment, and follow-up should be accompanied by the evaluation of muscle-specific biomarkers [7,66].

The occurrence of sarcopenia probably influences the production and release of myokines. Data from Park et al. [67] showed that pre-sarcopenic and sarcopenic older women presented a reduction in serum irisin as compared with non-sarcopenic women. Coelho-Junior et al. [32] proposed that myokine signaling is modified during sarcopenia, contributing to atrophy and reduced physical performance. On the other hand, muscle failure could lead to a reduction in the expression and production of myokines as a result of muscle stimulation, worsening muscle wasting, and the biological actions of myokines.

Several studies have proposed that the long-term benefits obtained from physical exercise may be, at least in part, promoted by the actions of myokines [68]; this is supported by the fact that the systemic concentrations of different myokines, such as IGF-1, irisin, and BDNF, are more elevated in older adults after exercise training. Considering these premises, if the endocrine role of muscle is not sufficiently stimulated by physical activity, the production and release of myokines may not be enough, contributing to the malfunction of many organs; with time, this biological scenario may lead to the development of frailty [59,69,70]. Studies focusing on BNDF in patients with sarcopenia are important. This myokine positively upregulates bone formation, due to the induction of osteoblast proliferation, and mineralization. The secretion of BDNF should be altered in patients with sarcopenic obesity, as this myokine is also an adipokine. It also modulates energy balance and is involved in the genesis of obesity. Patients in the late stages of diabetes mellitus show a reduction of BDNF in plasma, suggesting that its administration could be beneficial for this kind of patient [71,72,73].

Muscle protein breakdown and atrophy, along with the aging process, are related to oxidative stress, inflammation, and mitochondrial dysfunction. On the other hand, increased protein consumption and physical exercise counteract aging-related muscle loss. The expression of myokines occurs under both anabolic and catabolic situations, resulting in local and systemic effects [32]. For example, the concentrations of myostatin were independently related to muscle wasting and inversely related to body skeletal muscle mass in patients with chronic obstructive pulmonary disease and predicted one-year mortality in patients on hemodialysis [74,75,76].

The modifications observed in sarcopenia include elevated activity of the proteasomal pathway and increased sarcolemmal permeability, which may promote the secretion of myokines. The expression of bone morphogenetic factor-7 (BMP-7) and irisin are reduced in aged animals, but they are restored through physical exercise. BMP-7 stabilizes the neuromuscular junctions, which decline in number and function with aging. Furthermore, this molecule induces hypertrophy in animals through SMAD-1/5-mediated activation of the mTOR pathway. No modifications were observed in the levels of myostatin, but impairment is seen in the production of FGF-21 [14,77]. This myokine may also be produced by the liver, and we must remember that there is crosstalk between liver, muscle, and adipose tissues [78].

Studies have shown the effects of an IL-6-like cytokine in animals undergoing myocardial infarction. Contradictory studies show the beneficial role of this IL-6-like cytokine as well as its pro-cachectic action [79]. In aged animals, authors observed decreased levels of IL-15, which is thought to be an anabolic factor in skeletal muscle. Further actions are related to bone remodeling due to the stimulation of pre-osteoclast differentiation. The increase in the levels of IL-15 following resistance exercise induces an increase in bone mass and a decrease in fat mass, indicating that IL-15 could affect both fat mass and bone metabolism. During cachexia and sarcopenia, it can reduce the rate of protein degradation, which highlights the therapeutic importance of this cytokine [58,80].

**Figure 2:**
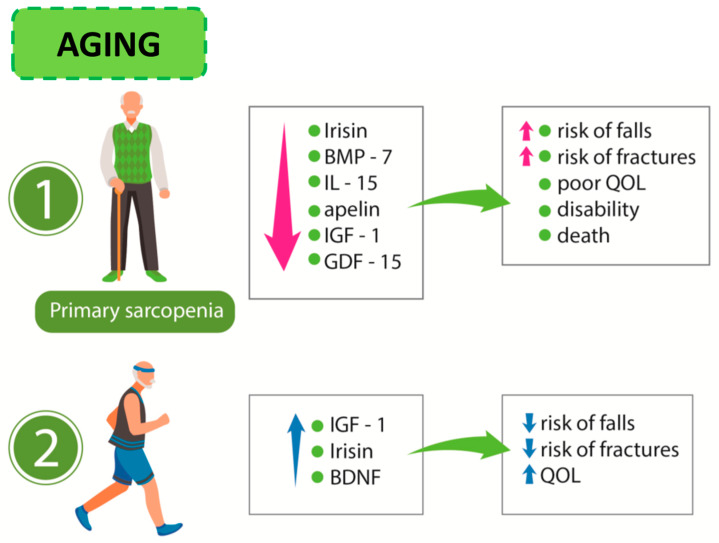
Aging process: (1) sarcopenia and its consequences and (2) influence of exercise on the release of myokines. BMP-7: bone morphogenetic factor-7; IL-15: interleukin-15; IGF-1: insulin-like growth factor-1; GDF-15: growth/differentiation factor-15 BDNF: brain-derived neurotrophic factor; QoL: quality of life.

In muscle wasting, elevated levels of growth/differentiation factor-15 (GDF-15), which is supposed to play a pro-cachectic role, are observed [81]. Figure 2 presents the relationship linking sarcopenia, myokine release, and effects of exercise training in the elderly.

Son et al. [82] suggested that both irisin and SPARC could work as therapeutic mediators to avoid muscle atrophy and sarcopenia.

### 2.3. Myokines, Sarcopenia, and Cardiovascular Diseases

Despite countless advances in medicine and correlated areas, the management of chronic disease is a challenge for professionals. Aging leads to physiological modifications in body composition, affecting disease outcomes and amplification of the burden to both public and private healthcare. As pointed out above, the physical adjustments related to age involve, for example, sarcopenia [83,84].

The aging process is associated with impairments in cardiovascular function, muscular strength, and cognitive ability. Mitochondrial dysfunction, reduction in protein synthesis and peroxisome PGC-1α levels, as well as their consequences such as protein degradation, atrophy, denervation, a shift of fatty acid oxidation to higher dependence on carbohydrates result in cardiomyopathy and other cardiovascular complications. The loss of muscle mass in individuals with chronic heart disease increases the risk of death [85].

Many studies bring to light that sarcopenia shares several pathophysiologic aspects with heart diseases in older adults. Sarcopenia is independently related to prevalent cardiovascular disorders, such as myocardial infarction, congestive heart failure, atrial fibrillation, atherosclerosis, and related risk factors [86,87].

In a cross-sectional study [67], it was shown that there is a significant impact of sarcopenia on the occurrence of stroke in elderly individuals. In another study with 316 elderly patients with stroke, Nozoe et al. [88] showed that the appearance of pre-stroke sarcopenia is an independent predictor of moderate to severe stroke.

Anaszewicz et al. [89] in a cohort study showed that patients with atrial fibrillation had a higher body mass index, larger waist circumference and visceral adiposity, higher values for fat and fat-free mass, and reduced percentage of skeletal muscle mass, indicating a higher prevalence of obesity and sarcopenia in these patients.

In a cross-sectional study, Santana et al. [90] investigated the value of sarcopenia per se and sarcopenia related to obesity as prognostic predictors of coronary heart disease. They found that sarcopenia and sarcopenic obesity was present in 64.6% and 35.4%, respectively, of elderly patients with acute myocardial infarction (AMI), and sarcopenia was associated with a higher cardiovascular risk score in AMI.

**Figure 3:**
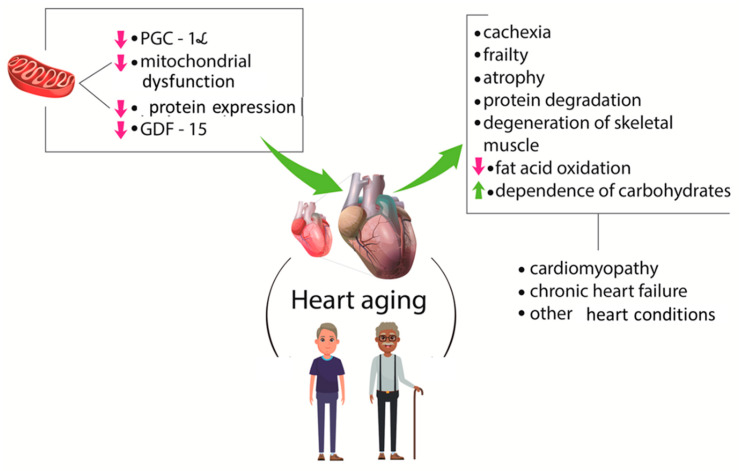
The relationship between sarcopenia and cardiovascular diseases. The aging process is related to mitochondrial dysfunction and reduction in protein synthesis and PGC-1α and GDF-15 levels. These factors are linked to atrophy, denervation, frailty, and modifications in lipid and carbohydrates metabolism that are associated with heart problems. PGC-1α: peroxisome proliferator-activated receptor gamma coactivator-1 alpha.

According to Nakajima et al. [4], GDF-15 levels are related to several pathophysiological conditions, including heart failure and cachexia. These authors studied the presence of this factor in cardiovascular surgery patients and found that preoperative GDF-15 levels are related to muscle wasting. For these reasons, the authors suggested that GDF-15 could be used as a novel biomarker for the identification of patients at high risk for muscle wasting before cardiovascular surgery. Figure 3 shows some consequences of the aging process and CVD.

On the other hand, the presence of chronic heart failure (CHF stage D) can be associated with the occurrence of sarcopenia, since it can lead to a reduction in muscle mass due to malnutrition, hormonal changes, inflammatory and oxidative processes, autophagy, and apoptosis. Also, CHF can aggravate some outcomes related to sarcopenia, including osteoporosis, falls, cachexia, frailty, hospitalization, and death [9].

Sarcopenic obesity can contribute to the morbidity and mortality of chronic diseases, including CVD. These findings are corroborated by a study of 664 cardiovascular surgery patients (over 60 years old) who underwent a preoperative evaluation of sarcopenia. The results of this study showed that sarcopenic obesity is closely associated with an increased risk of mortality in this group of patients [91].

A sedentary lifestyle hastens the aging process, putting an individual at significant risk for developing sarcopenia, metabolic syndrome, and CVD [92,93]. Increased adipose tissue resulting from the decrease in muscle mass in patients with sarcopenia may also be involved in the pathology of CVD, since there is a relevant increase in the production of pro-inflammatory cytokines. On the other hand, the decrease in muscle mass is accompanied by a reduction in the release of many myokines.

The above studies show a close relationship between the presence of sarcopenia and morbidity/mortality from cardiovascular complications. The question that needs to be answered is how much myokines in sarcopenic patients influence this outcome.

Furthermore, the meta-analysis performed by Guo et al. [94] of seven case–control studies showed that the concentrations of irisin were lower in patients with coronary artery disease or atherosclerosis compared with healthy controls.

The efficiency of skeletal muscle depends on the complex balance that should occur between the anabolic and catabolic pathways. This homeostasis deteriorates with aging, resulting in an age-related reduction in muscle quantity and quality. This condition is related to several systemic complications that occur for many reasons, including the modification in the pattern of synthesis and release of myokines. Physical inactivity and reduction in muscle mass lead to an increase in visceral fat deposition—culminating in an imbalance between anti- and pro-inflammatory status—supporting the vicious circle of sarcopenia and increasing fat mass and cardiovascular complications. The low-grade and chronic inflammatory scenario itself contributes to an imbalance in glucose and lipid metabolism, endothelial dysfunction, CVD, and sarcopenia [26,95,96,97].

The association of geriatric syndromes such as frailty, delirium, polypharmacy, multimorbidity, and sarcopenia can be seen as a continuum, distinct from an acute event such as AMI. These data are crucial because they can imply an increase in hospital time, health system costs, and mortality [98].

As we already briefly discussed, several myokines have metabolic actions associated with the prevention of CVD. Myostatin has local actions by inhibiting muscle hypertrophy and also acts remotely on adipose tissue. IL-6 may interfere with muscle triglycerides oxidation and with glycemia, since it stimulates the translocation of GLUT4 to the membrane improving insulin sensitivity in skeletal muscle. Furthermore, this myokine can stimulate lipolysis in the adipose tissue and lipid mobilization during exercise. IL15 is also reported to modulate the metabolic function [99].

The increase of energy expenditure promoted by irisin, through PPAR-α-dependent signaling, improves insulin sensitivity and reduces hepatic steatosis by upregulating FGF21. Together, these effects can independently lead to a reduction in body weight and adiposity in an obese mouse model. In human muscle, the expression of the fibronectin type III domain-containing protein 5 (FNDC5) gene, which encodes a precursor of irisin, is associated with the practice of physical activity and the increase in skeletal muscle mass [96,100,101].

Irisin also plays a role in the development and progression of arterial hypertension, diabetes mellitus, and chronic kidney disease. Irisin is associated with reduced blood pressure through nitric oxide-dependent pathways in animal models. Reduced levels of irisin are observed in diabetic patients when compared to non-diabetics [102].

FGF-21 shows anti-diabetic action in animal models through the stimulation of glucose transport in the adipose tissue. It also stimulates lipolysis and thermogenesis in brown adipose tissue, increases energy expenditure, and improves insulin sensitivity in response to exercise [103,104].

Hypertrophy associated with resistance training leads to augmented production of follistatin-like 1, which is capable of improving vascular injury, endothelial function, and myocardial ischemic damage [105].

Myonectin modulates anti-inflammatory and anti-apoptotic signaling cascades in the myocardium and can be a cardio-protective myokine. This could explain, at least in part, cardiovascular benefits produced by endurance exercises [51].

BDNF levels are reduced in heart failure patients and are positively correlated with the peak of oxygen uptake (VO_2_). Furthermore, reduced serum levels of BDNF were associated with all-cause cardiac death and readmission in heart failure patients, suggesting that this myokine could be a less invasive biomarker that shows the severity of heart failure and can work as a predictor of prognosis in these patients [106].

With the above considerations, we can make the hypothesis that, in the rehabilitation process of sarcopenic individuals, physical exercise could increase the expression of myokines, and then these molecules could positively interfere with the morbidity and mortality resulting from cardiovascular diseases.

These results make us wonder whether sarcopenic patients tend to produce less irisin and therefore have a higher chance of cardiovascular complications. On the other hand, patients with cardiac complications tend to express less irisin and tend to develop sarcopenia during hospitalization after surgery.

We are still very far from elucidating how much different myokines influence cardiovascular complications, osteoporosis, and sarcopenia. Perhaps, we are even further from knowing the relationship between these elements. When this knowledge comes to light, we may have better means of prevention and therapeutic management for elderly patients undergoing procedures related to cardiovascular events. It is also important to note that the development of immunobiology and other drugs that have myokines as therapeutic targets depends on a better understanding of the metabolic effects of myokines on skeletal muscle, bone, liver, and adipose tissue and of how these organs interconnect. Another gap to be explored would be to analyze the expression of myokines in sarcopenia not associated with aging. For this intent, we need to improve our understanding of the kinetics of muscle loss in different people and circumstances such as aging, disease, malnutrition, and a sedentary lifestyle.

Furthermore, although we have the answer as to how to prevent sarcopenia, we still have a long way to go in order to properly manage the sarcopenic patient or even seek strategies to reduce its progression. This pathway undoubtedly involves a better understanding of myokines and how to stimulate or, in some cases, block the synthesis of these factors.

To the best of our knowledge, this is the first time that the strong association of myokines, sarcopenia, and cardiovascular diseases has been shown.

## 3. Conclusions

Myokines are protagonists in a complex condition termed sarcopenia, which is associated with adverse clinical outcomes in patients with CVD. The discovery of new pathways and the link between myokines and CVD remain a cornerstone toward multifaceted interventions and perhaps the minimization of the damage resulting from muscle loss induced by factors such as atherosclerosis. Beyond that, there is still a long way to go before we can completely understand these interconnections and the plethora of possible roles of myokines that could help in the development of new therapeutic approaches for several harmful conditions, such as sarcopenia and CVD.
